# Role of obesity in a randomized placebo-controlled trial of difluoromethylornithine (DFMO) + sulindac for the prevention of sporadic colorectal adenomas

**DOI:** 10.1007/s10552-012-0051-6

**Published:** 2012-08-21

**Authors:** Jason A. Zell, Bruce S. Lin, Nikki Madson, Christine E. McLaren, Eugene W. Gerner, Frank L. Meyskens

**Affiliations:** 1 Chao Family Comprehensive Cancer Center, University of California, Irvine, CA 92697 USA; 2 Division of Hematology/Oncology, Department of Medicine, University of California, Irvine, CA 92697 USA; 3 Department of Epidemiology, University of California, Irvine, CA 92697 USA; 4 Division of Human Genetics and Metabolism, Department of Pediatrics, University of California, Irvine, CA 92697 USA; 5 Department of Cell Biology and Anatomy, College of Medicine, Tucson, AZ 85724 USA; 6 Gastrointestinal Cancer Program, Arizona Cancer Center, Tucson, AZ 85724 USA; 7 Department of Biological Sciences, University of California, Irvine, CA 92697 USA; 8 Division of Hematology/Oncology, University of California Irvine Medical Center, 101 The City Drive South, Orange, CA 92868 USA

**Keywords:** Body mass index, BMI, Colorectal adenoma, Chemoprevention, Difluoromethylornithine, Obesity, Sulindac

## Abstract

**Background:**

Chemoprevention with the polyamine-inhibitory regimen difluoromethylornithine (DFMO) + sulindac markedly reduces risk of recurrent adenoma in colorectal adenoma patients. Obesity is associated with risk of colorectal adenoma and colorectal cancer. This study investigates how obesity influences risk of recurrent adenoma after prolonged treatment with DFMO + sulindac versus placebo.

**Methods:**

Our analysis included subjects enrolled in the phase III colorectal adenoma prevention clinical trial investigating DFMO + sulindac versus placebo. Patients were classified by obesity (body mass index, BMI ≥ 30 kg/m^2^) status at baseline. Pearson χ^2^ statistic and Mann–Whitney *U* test were used to compare baseline characteristics, including rectal tissue polyamine levels. Log-binomial regression analysis was used to determine the risk ratio (RR) of recurrent adenomas, adjusted for covariates and an interaction term for obesity and treatment.

**Results:**

The final analytic cohort was comprised of 267 patients. In separate regression models, the risk of adenoma recurrence after treatment compared to placebo was similar for obese (RR = 0.32, 95 % CI 15–71) and non-obese patients (RR = 0.27, 95 % CI 15–49). No significant interaction was detected between obesity, treatment, and risk of colorectal adenoma in the full regression model (*p*_interaction_ = 0.91).

**Conclusions:**

Obesity does not substantially modify the colorectal adenoma risk reduction ascribed to DFMO + sulindac versus placebo.

## Introduction

Polyamines are naturally occurring substances that, in excess, are associated with colorectal carcinogenesis in animal models [1]. In a randomized phase III trial of colorectal adenoma (CRA) patients, the risk of CRA recurrence was decreased by 70 % after 3 years of treatment with the polyamine-inhibitory regimen difluoromethylornithine (DFMO) + sulindac compared to placebo. These effects were shown to occur via polyamine-dependent processes [2], with differential treatment outcomes based on genetic polymorphism of *ODC1* (*ornithine decarboxylase*-*1*)—a key regulatory gene of polyamine metabolism [3]. Numerous observational studies have reported associations between obesity and risk of CRA or colorectal cancer (CRC), with variable reporting of obesity-associated risks based on gender, colorectal subsite, and adenoma characteristics (e.g., number, size, histology, and high-risk features) [4–9]. Polyamine metabolism and its inhibition have been associated with increased adipose tissue and weight gain in animal models. Murine experiments reveal that polyamine inhibition via knockout of *spermidine spermine acetyltransferase*, *SSAT* (a gene encoding SSAT, which is responsible for polyamine acetylation and subsequent cellular polyamine export), results in deceased fatty acid catabolism, increased tissue adipose content, and increased weight gain [10]. These findings indicate potential links between obesity and polyamine inhibition in humans. Polyamines are derived from dietary sources in humans (meats, corn, peas, grapefruit juice) [11]. However, potential relationships between energy balance and polyamine-related colorectal carcinogenesis have not been described in humans. Here, we examine whether obesity modifies the adenoma risk reduction conferred by polyamine-inhibitory treatment among colorectal adenoma patients.

## Materials and methods

The parent study was a randomized, double-blind, placebo-controlled multi-site clinical trial to determine the effect of DFMO + sulindac on CRA recurrence [12]. Eligible patients were between 40 and 80 years of age with a history of ≥1 resected adenoma (≥3 mm) within 5 years before study entry. All patients underwent colonoscopy and removal of any preexisting polyps within 6 months of study entry. Body mass index (BMI) was calculated with direct measurement of each participant’s height and weight. The proportion of obese (BMI ≥ 30 kg/m^2^) versus non-obese (BMI < 30 kg/m^2^) patients was similar between screened (34 % vs. 66 %), enrolled (33 % vs. 67 %), and non-enrolled participants (40 % vs. 60 %). The study was approved by the University of California-Irvine Institutional Review Board (# 2002–2261).

In the parent trial, a total of 375 subjects were randomized; planned treatment duration was 36 months. At the second interim analysis, the study was halted by the Data Safety and Monitoring Board since clinical efficacy endpoints were achieved. For the present analysis, data were obtained from all 267 patients completing end-of-study colonoscopies. The primary objective of the present study was to determine whether obesity modifies the effect of DFMO + sulindac (vs. placebo) on CRA recurrence. Two categories were defined by BMI status including a non-obese and obese group. For the comparisons of baseline characteristics of patients in the obese versus non-obese groups, dichotomous variables were created to represent factors previously associated with increased risk of advanced metachronous adenoma. These included the presence of proximal (right-sided) lesions, defined as those in the transverse colon, right colon, and cecum; large adenomas, defined as ≥10 mm in size; multiple adenomas (3 or more); adenomas with advanced histology (i.e., villous or tubulovillous features, high-grade dysplasia, and carcinoma-in situ); and high-risk lesions, which included either advanced adenomas, multiple adenomas, or those >10 mm in size.

## Statistical analysis

Comparisons of demographic, clinical, and pathological variables were performed using Pearson χ^2^ statistic for nominal variables and Mann–Whitney *U* test for continuous variables that were not normally distributed: age, tissue polyamines, and number of adenomas. The risk ratio of development of any recurrent adenoma was assessed by log-binomial regression, with adjustment for treatment group, obesity, age, aspirin use, and a term representing the interaction of obesity and treatment group. Aspirin use was included in the model as this was a stratification factor in the parent trial and used in the primary efficacy analyses of that trial [12]. Age was included in the full regression model (containing obese and non-obese patients) due to the baseline differences observed in the obese versus non-obese groups and excluded from the multivariate regression models that were restricted to either the obese group or non-obese group. Seventy-two patients had developed metachronous adenomas in the final dataset. Statistical analyses were conducted using SAS 9.2 statistical software (SAS Inc., Cary, NC).

## Results

Baseline characteristics of the final analytic cohort are presented in Table 1. The median age of all participants was 60.8 years. The median BMI was 28.8 kg/m^2^, with a range of 17.0–52.4 kg/m^2^. The non-obese group consisted of approximately twice as many patients as the obese group. The obese group was significantly younger than the non-obese group: 59.0 versus 61.9 years (*p* = 0.004). No significant differences were observed between obesity groups for gender, ethnicity, aspirin use, treatment received, or baseline rectal tissue polyamine contents. Significant baseline differences in adenoma characteristics between obese and non-obese patients were observed.

**Table 1:** Clinicopathologic baseline characteristics of the final analytic cohort

	All (*n* = 267)	BMI < 30 (*n* = 181)	BMI ≥ 30 (*n* = 86)	p*
*Age (years)*				
Median	60.8	61.9	59.0	0.004^a^
Range	41.4–78.8	41.4–78.8	42.4–73.8	
*Sex*				
Male	202 (75.7 %)	138 (76.2 %)	64 (74.4 %)	0.74
Female	65 (24.3 %)	43 (23.8 %)	22 (25.6 %)	
*Ethnicity*				
Asian/Pacific Islander	12 (4.5 %)	11 (6.1 %)	1 (1.1 %)	0.14
Black	8 (3.0 %)	5 (2.8 %)	3 (3.5 %)	
Hispanic	19 (7.1 %)	9 (5.0 %)	10 (11.6 %)	
White	224 (83.9 %)	153 (84.5 %)	71 (82.6 %)	
Other	4 (1.5 %)	3 (1.7 %)	1 (1.1 %)	
*Aspirin use*				
Yes	103 (38.6 %)	74 (40.9 %)	29 (33.7 %)	0.26
No	164 (61.4 %)	107 (59.1 %)	57 (66.3 %)	
*BMI (kg/m* ^*2*^ *)*				
Median	28.8	–	–	–
Range	17.0–52.4	–	–	
95 % CI	21.9–39.3	–	–	
*Treatment*				
DFMO/sulindac	138 (51.7 %)	95 (52.5 %)	43 (50.0 %)	0.70
Placebo	129 (49.3 %)	86 (47.5 %)	43 (50.0 %)	
*Tissue polyamines* ^*b*^ *(nmol/mg)* ^*c*^				
Putrescine				
Median	0.49	0.5	0.49	0.58^a^
Range	0.01–5.29	0.01–5.29	0.01–3.27	
Spermidine				
Median	2.06	2.07	2.05	0.93^a^
Range	0.76–11.45	0.76–9.18	1.05–11.45	
Spermine				
Median	7.07	7.07	7.12	0.43^a^
Range	2.29–34.10	2.29–28.31	3.88–34.10	
Spermidine:spermine Ratio				
Median	0.3	0.3	0.29	0.33^a^
Range	0.19–0.98	0.19–0.98	0.20–0.76	
*Number of adenomas* ^*b*^				
Mean	2.4 (± 2.0 SD)	2.2 (± 1.6 SD)	3.0 (± 2.6 SD)	0.006
Median	2	2	2	
95 % CI	1–6	1–6	1–8	
*Adenoma size (mm)*				
<10	183 (68.5 %)	136 (75.1 %)	47 (54.6 %)	0.0008
≥10	84 (31.5 %)	45 (24.9 %)	39 (45.4 %)	
*Multiple adenomas* ^*b*^				
<3	182 (68.9 %)	132 (73.7 %)	50 (58.8 %)	0.01
≥3	82 (31.1 %)	47 (26.3 %)	35 (41.2 %)	
*Advanced adenoma histology* ^*d*^				
Yes	46 (17.2 %)	24 (13.3 %)	22 (25.6 %)	0.013
No	221 (82.8 %)	157 (86.7 %)	64 (74.4 %)	
*Location* ^*e*^				
Proximal^f^	99 (37.2 %)	78 (43.3 %)	21 (24.4 %)	0.003
Distal^g^	167 (62.8 %)	102 (56.7 %)	65 (75.6 %)	
*High-risk adenomas* ^*h*^				
Yes	144 (53.9 %)	82 (45.3 %)	62 (72.1 %)	<0.0001
No	123 (46.1 %)	99 (54.7 %)	24 (27.9 %)	

* *p* values indicate comparisons between the obese and non-obese groups

^a^Mann–Whitney *U* test

^b^Data missing from three patients

^c^nmol polyamine per milligram protein

^d^Includes adenomas with villous or tubulovillous features, high-grade dysplasia, or carcinoma-in situ

^e^Data missing from one patient

^f^Includes the cecum, right colon, and transverse colon

^g^Includes the left colon and rectum

^h^Includes adenomas >1 cm in size, multiple adenomas (3 or more at baseline), or those with the following histologic characteristics: villous or tubulovillous features, high-grade dysplasia, or carcinoma-in situ

Among 86 obese patients, 23 patients had recurrent adenomas at the end-of-study, including 6 recurrences among 43 patients (14 %) in the DFMO + sulindac group, and 17 recurrences among 43 patients (40 %) in the placebo group. The risk ratio of adenoma recurrence after treatment (compared to placebo, as a referent group) among obese patients was 0.32, 95 % confidence interval, CI = 0.15–0.71 (Table 2). Among the 181 non-obese patients, 49 patients had recurrent adenomas at the end-of-study, including 11 recurrences among 95 patients (12 %) in the DFMO + sulindac group, and 38 recurrences among 86 patients (44 %) in the placebo group. Among non-obese patients, the risk ratio of adenoma recurrence after treatment (compared to placebo, as a referent group) was 0.27, with 95 % CI = 0.15–0.49 (Table 2). In the full regression model including all 267 subjects, with adjustment for treatment group, obesity, age, aspirin use, and a term representing the interaction of obesity and treatment, no significant interaction was noted between treatment and obesity with regard to adenoma recurrence (*p* = 0.91). Main effects for obesity were not significant in the full risk models when analyzed as a dichotomous variable (obese vs. non-obese): RR = 1.20, 95 % CI 0.72–2.02; *p* = 0.49, or as a continuous variable (BMI): RR = 1.01, 95 % CI 0.98–1.05; *p* = 0.45. Further analyses based on risk of multiple or advanced adenomas were not performed due to a low number of events in the treatment group.

**Table 2:** Colorectal adenoma recurrence risk* after treatment with DFMO + sulindac versus placebo, by obesity status at baseline

	Non-obese patients (*n* = 181)			Obese patients (*n* = 86)		
	*n*	Risk ratio (95 % confidence interval)	*p*	*n*	Risk ratio (95 % confidence interval)	*p*
*Recurrent adenoma events*						
Any adenoma	49	0.27 (0.15–0.49)	<0.0001	23	0.32 (0.15–0.71)	0.005

Risk ratios indicate the effect of DFMO + sulindac compared with placebo (as a referent value) on recurrent colorectal adenomas

* Relative risk estimation by log-binomial regression. Likelihood ratio test *p* values are reported. Risk ratios indicate risk of metachronous adenoma after treatment with DFMO + sulindac versus placebo (referent group). All risk ratios are adjusted for aspirin intake

## Discussion

Here, we report that obesity does not modify the CRA risk reduction previously ascribed to DFMO + sulindac versus placebo. We observed a 68 % reduction in recurrent CRA among obese patients (vs. 73 % reduction among non-obese patients) after prolonged administration of DFMO + sulindac compared with placebo. Obesity itself was not found to be associated with recurrent CRAs, a finding congruent with some—but not all previous reports [4, 13, 14]. Obesity was associated with several baseline adenoma characteristics that are risk factors for advanced adenoma recurrence; however, the trial was not designed to prospectively stratify patients by obese status in the randomization process. In contrast to results from mouse model experiments noting associations between obesity and polyamine levels [10], we did not observe any differences in rectal tissue polyamine levels among trial participants based on obesity status at baseline (Table 1).

Inconsistencies in the literature regarding associations between obesity and risk of CRA or CRC may be partly related to obesity definitions. While many studies have utilized BMI as an indicator of obesity, other measures of obesity are studied (e.g., waist circumference, waist-to-hip ratio, visceral adipose tissue). Abdominal obesity has been shown to be linked to insulin resistance and hyperinsulinemia [15], which has been suggested to underlie the association of obesity and CRA. In vitro studies have shown that insulin promotes cell growth in colonic mucosa and in colon carcinoma cells [16]. Epidemiological data indicate that metabolic syndrome, a cluster of metabolic abnormalities including insulin resistance and central obesity, as well as insulin-dependent Type II diabetes, are biological risk factors for the development of CRAs and CRCs [17]. Insulin levels and insulin-like growth factor-I are positively associated with CRA incidence, especially advanced adenomas. Interestingly, the same study found no association between visceral adipose tissue and BMI and adenoma risk [13]. Therefore, obesity may be acting as a surrogate risk factor not only for hyperinsulinemia, but also for other potential underlying factors such as low physical activity or high-risk dietary patterns [18]. As these factors typically do not exist in isolation, it remains difficult to determine to what degree each of these factors plays a role in colorectal adenoma and cancer risk.

A recent Japanese study investigated how the adipokines mediate associations between obesity and CRC [19]. An inverse association between adiponectin level and CRA was found, whereas a positive association of leptin was noted. Adiponectin may exert anticarcinogenic effects on the large intestine by interfering with leptin, whereas leptin could conversely exert carcinogenic effects under conditions of lower adiponectin levels. Since adipokines play an important role in insulin resistance [20], future studies on the interactions between adipokines and the insulin pathway may better elucidate underlying mechanisms. A National Cancer Institute-sponsored multi-institutional phase IIa clinical biomarker trial investigating adipokines and other relevant biomarkers pre- and post-metformin treatment in obese CRA patients is currently ongoing at the University of California Irvine, with results anticipated in 2013 [21].

Several limitations of this study must be acknowledged. Our analysis was performed using data from the controlled setting of a phase III trial with a relatively small sample size. The recurrence analysis was limited by the overwhelming effect of treatment in approximately half of the study population (i.e., some of the effects of obesity, if present, may have been minimized). Additionally, we were not able to test other measures of obesity (e.g., central obesity or percentage body fat), which may have resulted in different outcomes, and did not account for other relevant lifestyle or hormonal factors. It is possible that differences in drug metabolism may occur based on the volume of distribution (which is increased in patients with large amounts of adipose tissue) or under-dosing due to a particular fixed-dose regimen utilized here. For example, in oncology, drug dosing of obese patients has been identified as a potential factor for the observed poor outcomes among obese cancer patients. In obese cancer patients, it is believed that fixed drug dosing or dose “capping” (i.e., limiting the body surface area to a pre-specified maximum number) may result in inadequate drug delivery [22]. It is important to note that the tissue polyamine contents evaluated here refer to steady-state levels of specifically rectal mucosal polyamine contents. Differences in adipose tissue polyamine contents were not addressed in our study, which represents a limitation of the analysis. Additionally, we did not examine total polyamine flux—which may be important in understanding polyamine effects on metabolism.

Our analysis suggests that obesity does not substantially modify CRA risk reduction after treatment with DFMO + sulindac compared with placebo. This has implications for therapeutic prevention of CRAs, since a key goal of cancer prevention clinical trials is to refine the risk:benefit, and risk:risk profile of chemopreventive agents. The large risk reduction afforded to CRA patients receiving DFMO + sulindac as compared to placebo in the parent trial appears to occur regardless of whether or not patients are obese. Potential benefits of lifestyle modifications on colorectal carcinogenesis in general (including control of obesity, increasing physical activity, and specific dietary modifications) are clearly relevant and beyond this scope of this manuscript.

## Participating institutions and investigators

Chao Family Comprehensive Cancer Center, University of California Irvine, CA: Frank L. Meyskens Jr, MD (Principal Investigator); Gregory Albers, MD; Sharon Fujikawa-Brooks, PhD; Philip M. Carpenter, MD; Daniel L. Gillen, PhD; Christine E. McLaren, PhD; Daniel Pelot, MD. Arizona Cancer Center, University of Arizona, AZ: Eugene W. Gerner, PhD; Steven Goldschmid, MD; Peter Lance, MD. Denver Department of Veteran Affairs Medical Center and University of Colorado: Dennis J. Ahnen, MD. Department of Veterans Affairs Long Beach Healthcare System, CA: Jayashri Kidao, MD. Kaiser Permanente, Sacramento, CA: Michael J. Lawson, MD. Loma Linda University, CA: John McCracken, MD. Department of Veterans Affairs Loma Linda Healthcare System, CA: Ronald Griffen, MD. University of Michigan, Ann Arbor, CA: D. Kim Turgeon, MD. University of Kansas, Kansas City, CA: Curt H. Hagedorn, MD.
